# Deciphering Biological Processes at the Tick-Host Interface Opens New Strategies for Treatment of Human Diseases

**DOI:** 10.3389/fphys.2019.00830

**Published:** 2019-07-03

**Authors:** Iveta Štibrániová, Pavlína Bartíková, Viera Holíková, Mária Kazimírová

**Affiliations:** ^1^Biomedical Research Center, Institute of Virology, Slovak Academy of Sciences, Bratislava, Slovakia; ^2^Institute of Zoology, Slovak Academy of Sciences, Bratislava, Slovakia

**Keywords:** tick saliva, immunomodulation, host defense, pharmacological molecules, immunological disorders

## Abstract

Ticks are obligatory blood-feeding ectoparasites, causing blood loss and skin damage in their hosts. In addition, ticks also transmit a number of various pathogenic microorganisms that cause serious diseases in humans and animals. Ticks evolved a wide array of salivary bioactive compounds that, upon injection into the host skin, inhibit or modulate host reactions such as hemostasis, inflammation and wound healing. Modulation of the tick attachment site in the host skin involves mainly molecules which affect physiological processes orchestrated by cytokines, chemokines and growth factors. Suppressing host defense reactions is crucial for tick survival and reproduction. Furthermore, pharmacologically active compounds in tick saliva have a promising therapeutic potential for treatment of some human diseases connected with disorders in hemostasis and immune system. These disorders are often associated to alterations in signaling pathways and dysregulation or overexpression of specific cytokines which, in turn, affect mechanisms of angiogenesis, cell motility and cytoskeletal regulation. Moreover, tick salivary molecules were found to exert cytotoxic and cytolytic effects on various tumor cells and have anti-angiogenic properties. Elucidation of the mode of action of tick bioactive molecules on the regulation of cell processes in their mammalian hosts could provide new tools for understanding the complex changes leading to immune disorders and cancer. Tick bioactive molecules may also be exploited as new pharmacological inhibitors of the signaling pathways of cytokines and thus help alleviate patient discomfort and increase patient survival. We review the current knowledge about tick salivary peptides and proteins that have been identified and functionally characterized in *in vitro* and/or *in vivo* models and their therapeutic perspective.

## Introduction

For centuries, humans have exploited arthropods (insects, scorpions, spiders, centipedes, ticks) and other invertebrates (leeches, hookworms, snails, etc.) and their products (honey, royal jelly, venom, propolis, etc.) or biologically active substances derived from them as valuable resources to treat various diseases (Cherniack, 2010, 2011; Chen et al., 2018; Koh et al., 2018). Their properties range from immunomodulatory, analgesic, anti-bacterial, anti-coagulant, anticancer, diuretic and anesthetic to anti-rheumatic. Many human cultures, especially in Asia, Africa and South America, have used arthropods in traditional medicine and highly appreciated their therapeutic potential. In some cases, whole organisms, in others individual ingredients have been used. The capabilities of maggots (larvae of blowflies) feeding on necrotic tissues to heal wounds are among well-studied medical application of insects (maggot therapy) (Wolf and Hansson, 2003). Of other invertebrates, medicinal leeches have been applied, e.g., in reconstructive microsurgery, and in treatment of phlebitis (Eldor et al., 1996) or osteoarthritis (Michalsen et al., 2002). Anticoagulants designed based on their product, hirudin and its derivatives, have been used as antithrombotics (Markwardt, 2002; Chudzinski-Tavassi et al., 2018; Koh et al., 2018). Endoparasitic helminths have been used to cure inflammatory bowel diseases (Buning et al., 2008), and cantharidin, a derivate from blister beetles, has been applied for warts, molluscum contagiosum (Moed et al., 2001) and in anticancer therapy (Efferth et al., 2005; Liu and Chen, 2009). The enormous richness and diversity of arthropods, the wide range of biological activities exerted by their products, the use of some species and their products or derivatives as drugs against common and important diseases suggest that arthropods are a rich, and yet unexplored and unexploited source of potentially useful compounds for development of new therapeutic agents for modern medicine (Pemberton, 1999).

Among blood-feeding arthropods, ticks occupy a unique position. Their parasitic lifestyle resulted in development of a wide spectrum of evasive and disarming mechanisms of host defense reactions. Tick salivary glands produce and secrete into the feeding site in the host skin an impressive amount and variety of bioactive molecules modulating hemostatic, inflammatory and immune responses as well as wound healing. The composition of tick saliva is highly complex and changes through the feeding process. There are a few comprehensive and recent reviews on the composition and role of saliva in tick feeding and tick-host-pathogen interactions (Francischetti et al., 2009; Kotal et al., 2015; Blisnick et al., 2017; Šimo et al., 2017; Nuttall, 2018; Wikel, 2018). They all highlight the fact that only an integrated understanding of the physiological roles of tick bioactive molecules and their mode of action can elucidate complex changes in the mammalian hosts, leading to immune disorders and cancer, and disclose therapeutically valuable molecules. This review is focused on identified tick salivary peptides and proteins with known structure and function(s) which are promising candidates for development of drugs and their recombinant forms have been tested in disease models *in vitro* and/or *in vivo*. Non-peptide molecules (e.g., nucleosides, lipids) contained in tick saliva are beyond the scope of this paper.

## Ticks and Pharmacological Properties of Their Saliva

Ticks are blood feeders and bioactive compounds in their saliva have a promising therapeutic potential for treatment of some human diseases associated with hemostatic and immunological disorders (Mans, 2005; Hovius et al., 2008; Francischetti, 2010; Štibrániová et al., 2013; Sousa et al., 2015; Bonvin et al., 2016; Chudzinski-Tavassi et al., 2016, 2018; Chmelar et al., 2017; Murfin and Fikrig, 2017; Parizi et al., 2018). Tick salivary glands, a paired organ consisting of acini in grape-like clusters, produce saliva secreted by the feeding tick into the host primarily to enable blood feeding by suppressing local hemostatic and host immune responses (Francischetti et al., 2009; Kazimírová and Štibrániová, 2013; Wikel, 2013; Kotal et al., 2015; Šimo et al., 2017; Nuttall, 2018). Tick feeding, in addition, enables host infection with pathogenic microorganisms carried by ticks. Cutting-edge high-throughput technologies used during the last decade for studying composition and function of tick saliva have revealed its complexity (Ribeiro et al., 2006; Francischetti et al., 2011; Radulovič et al., 2014; Kotsyfakis et al., 2015; Tan et al., 2015; Xu et al., 2015; de Castro et al., 2016; Mans, 2016; Bonnet et al., 2018); not surprisingly, considering the tick’s biology and their parasitic lifestyle, i.e., strict hematophagy, short-term (soft ticks) to long-lasting feeding (hard ticks) on the vertebrate host, and broad spectrum of hosts. The composition of tick saliva is complex and changes with biological factors such as gender, developmental stage, feeding stage and/or the presence/absence of microorganisms, pathogenic as well non-pathogenic (Liu et al., 2014; Ayllón et al., 2015; Kotsyfakis et al., 2015; Yu et al., 2015; Bonnet et al., 2017, 2018).

Tick saliva is a mixture of proteins, peptides and non-peptide molecules that interfere with various components of hemostasis, wound healing, and both arms of the immune system of the vertebrate hosts, including enzymes, cytokines, complement, antibodies, cell signaling components, immune cells (Francischetti et al., 2009; Mans, 2016; Nuttall, 2018; Wikel, 2018). In addition, cytotoxic and cytolitic activities acting against different cell types, impairment of cancer cells migration and signaling pathways, as well as anti-angiogenic properties have been demonstrated for saliva of different hard tick species (Kazimirova et al., 2006; Poole et al., 2013; Holíková et al., 2018; Sousa et al., 2018; de Sá Jr. et al., 2019; Gradowski do Nascimento et al., 2019), showing that tick saliva is an important source for designing new anticancer drugs (Kazimírová, 2011; Sousa et al., 2015; Chudzinski-Tavassi et al., 2016). Proteinaceous components of the tick saliva are grouped into families like lipocalins, proteins with Kunitz type domain, metalloproteases, serpins, cystatins, basic-tail secreted proteins, small peptide inhibitors, some protein families unique to ticks, and proteins and peptides of unknown structure and function (Ribeiro et al., 2006; Francischetti et al., 2009; Chmelar et al., 2012). Interestingly, a high redundancy and multifunctionality of the tick salivary compounds has been revealed, whereby many of them can target multiple components of hemostasis and, in addition, also components of the immune system (Francischetti et al., 2009; Chmelar et al., 2016; Šimo et al., 2017).

## Protease Inhibitors

Transcriptome and proteome studies of tick salivary glands (SGs) discovered an enormous protein diversity and unique proteins belonging to novel protein families with unknown functions (Francischetti et al., 2011; Radulovič et al., 2014; Kotsyfakis et al., 2015; Mans, 2016; Esteves et al., 2017). Many of these proteins are differentially expressed during the feeding process (Mans et al., 2008; Kotsyfakis et al., 2015).

Enzyme activity inhibitors represent a very abundant group and include, among others, protease inhibitors containing the Kunitz domain, serine protease inhibitors (serpins), cysteine proteinase inhibitors (cystatins), peptides of the hirudin-like/madanin/variegin superfamily, and basic tail proteins (de Miranda Santos et al., 2004; Francischetti et al., 2005, 2009; Ribeiro et al., 2006; Garcia et al., 2014; Liu and Bonnet, 2014; Parizi et al., 2018).

### Kunitz Domain Containing Proteins

Members of the Kunitz domain family, the one of the larger protein families expressed in tick salivary glands, have been functionally characterized primarily as anti-hemostatic agents that block or inhibit host blood coagulation and/or platelet aggregation (Corral-Rodriguez et al., 2009; Chmelar et al., 2012; Parizi et al., 2018), but some of them have been found to display multiple functions, e.g., ixolaris (de Oliveira et al., 2012) or Amblyomin X (Chudzinski-Tavassi et al., 2010).

Hemostasis is the first line of defense against the tick bite and the first stage of wound healing. It comprises a series of physiological processes that stop bleeding at the site of vascular injury by formation of a hemostatic plug. Three major mechanisms are involved in hemostasis: (i) vasoconstriction – termination of bleeding from damaged blood vessels, (ii) coagulation – production of a fibrin clot, (iii) formation of a platelet plug. The enzymes in the coagulation cascade are activated through different pathways, depending on various endogenous and exogenous factors (Hoffman et al., 2009). Ticks have evolved various and effective countermeasures against the different mechanisms of the vertebrate hemostatic system and can target single or multiple host coagulation factors (Maritz-Olivier et al., 2007; Francischetti et al., 2009; Kazimírová et al., 2010; Chmelar et al., 2012; Šimo et al., 2017). Thrombin is the main target for majority of the identified tick anticoagulants (Table 1) and inhibition of thrombin generation is one of the main strategies to prevent thrombosis (Kazimírová et al., 2010; Chmelar et al., 2012). However, drugs that target other coagulation factors, e.g., factor Xa (FXa) would be an alternative treatment when thrombin generation has already occurred (Yeh et al., 2012). In spite of the wide range of different identified inhibitors derived from tick salivary glands, due to strict criteria for clinical use, only a limited number passed pre-clinical and clinical tests. For example, only preliminary validations of the tick anticoagulant peptide (TAP) has been performed *in vivo* using animal models, but TAP has never been tested in humans due to a slow onset of action and because its antigenicity, and a single study performed for ixolaris in a rat model awaits future validation (Maritz-Olivier et al., 2007). However, as tick anticoagulants bind specifically to their target molecules, they are important molecular tools to study and increase our understanding of the mechanisms of host blood coagulation. Examples include the mapping of thrombin exosites by ornithodorin derived from *Ornithodoros moubata* (van der Locht et al., 1996), understanding the prothrombinase complex formation by using ixolaris from *I. scapularis* (Monteiro et al., 2005), or characterization of the molecular mechanisms that maintain the procofactor state of circulating FV and the conversion of FV to active cofactor FVa by means of recombinant TIXC-5 from *I. scapularis* (Aleman and Wolberg, 2013; Schuijt et al., 2013). In addition, information on the structure and function of tick-derived anticoagulants can be used in designing synthetic peptides as a basis for development of novel drugs (Maritz-Olivier et al., 2007; Koh et al., 2018).

**TABLE 1:** Examples of tick salivary molecules of therapeutic interest in human diseases.

**Molecule**	**Main function: Target(s) Proposed drug**	**Tick species**	**References**
**Protease inhibitors**			
**Serine protease inhibitors - Kunitz domain containing proteins**			
TAP	Anticoagulant: FXa Antithrombotic	*Ornithodoros moubata*	Waxman et al., 1990; Corral-Rodriguez et al.,2009
Ornithodorin	Anticoagulant: thrombin Antithrombotic	*O. moubata*	van der Locht et al.,1996
Ixolaris	Anticoagulant: inhibitor of contact system proteins (VIIa/tissue factor-induced FX, FXa) Antithrombotic, antitumor (glioblastoma, melanoma)	*Ixodes scapularis*	Francischetti et al., 2002; Nazareth et al., 2006; Carneiro-Lobo et al., 2009; de Oliveira et al.,2012
Amblyomin-X	Anticoagulant: FXa, FVIIa/TF complex activity, prothombin conversion Antitumor, anti-angiogenetic	Amblyomma sculptum (formerly *A. cajennense*)	Batista et al., 2010; Drewes et al., 2012, 2015; Maria et al., 2013; Pacheco et al., 2014; Branco et al., 2016; Chudzinski-Tavassi et al.,2016
BmTI-A	Anticoagulant: inhibitor of contact system proteins, inhibits plasmin, elastase, and plasma kallikrein Inhibitor of wound healing and vessel formation Anti-angiogenetic	*Rhipicephalus. (Boophilus) microplus*	Tanaka et al., 1999; Soares et al.,2016
Ir-CPI	Anticoagulant: inhibitor of contact system proteins, blocks FXII, FXI, and kallikrein activation Antithrombotic	*Ixodes ricinus*	Decrem et al.,2009
TdPI	Immunomodulation: innate immune responses, inflammation, inhibitor of human skin β-tryptase Anti-inflammatory	*Rhipicephalus appendiculatus*	Paesen et al.,2007
Tryptogalinin	Immunomodulation: innate immune responses, inhibitor of human skin β-tryptase Anti-inflammatory	*I. scapularis*	Valdés et al.,2013
Haemangin	Wound healing, angiogenesis: inhibits angiogenesis and neovascularization Anti-angiogenetic	*Haemaphysalis longicornis*	Islam et al.,2009
Disagregin	Antiplatelet agent: inhibitor of glycoprotein IIb/IIIa (integrin αIIbβ3) Antithrombotic	*O. moubata*	Karczewski et al.,1994
**Serine protease inhibitors – Serpin domain family**			
Iris	Anticoagulant: thrombin, FXa Immunosuppression: pro-inflammatory cytokines Drug acting in regulation of TNF-alpha overexpression	*I. ricinus*	Leboulle et al., 2002; Prevot et al.,2009
IRS-2	Immunomodulation: innate immune responses – T cells, T17 cells, cathepsin G, chymase Treatment of autoimmune diseases	*I. ricinus*	Chmelar et al., 2011; Palenikova et al.,2015
**Cystatins (cysteine protease inhibitors)**			
Sialostatin L	Immunomodulation: acquired immune responses – cathepsin L and V, papain, dendritic cells maturation Immunosuppressive drug for asthma attack	*I. scapularis*	Kotsyfakis et al., 2006; Sa-Nunes et al., 2009; Horka et al., 2012; Klein et al., 2015; Lieskovská et al.,2015b
Sialostatin L2	Immunomodulation: acquired immune responses - cathepsin L, V and S, papain, interferon Anti-inflammatory	*I. scapularis*	Kotsyfakis et al., 2007; Chen et al., 2014; Lieskovská et al.,2015a, b
Iristatin	Immunomodulation: innate immune responses Immunotherapeutic	*I. ricinus*	Kotál et al.,2019
DsCystatin	Immunomodulation: cathepsin L and B, pro-inflammatory cytokines, TLR signaling pathway Anti-inflammatory	*Dermacentor silvarum*	Sun et al.,2018
**Hirudin-like/Madanin/Variegin superfamily**			
Variegin	Anticoagulant: thrombin Antithrombotic	*Amblyomma variegatum*	Koh et al., 2007, 2011; Kazimírová et al.,2015
Avathrin	Anticoagulant: thrombin Antithrombotic, coating of medical devices	*A. variegatum*	Iyer et al., 2017, 2019|
Sculptin	Anticoagulant: thrombin Antithrombotic	*A. sculptum*	Iqbal et al.,2017
Madanin-1, 2, chimadanin	Anticoagulant: thrombin Antithrombotic	*H. longicornis*	Iwanaga et al., 2003; Thompson et al.,2017
Hyalomin 1	Anticoagulant: thrombin Antithrombotic	*Hyalomma marginatum*	Jablonka et al.,2015
Basic tail-secreted proteins			
Salp14	Anticoagulant: FXa Antithrombotic	*I. scapularis*	Narasimhan et al.,2002
Ixonnexin	Modulator of haemostasis: promotes fibrinolysis Antithrombotic	*I. scapularis*	Assumpção et al.,2018
**Lipocalins**			
OmCI	Complement: inhibitor of C5 activation Coversin: second-generation complement inhibitor; acute and chronic inflammation	*O. moubata*	Nunn et al., 2005; Roversi et al., 2007; Barratt-Due et al.,2011
Moubatin and other lipocalins of the moubatin clade from soft ticks	Antiplatelet agent: inhibition of platelet aggregation, binds to thromboxane A2 Antithrombotic	*O. moubata*	Waxman and Connolly, 1993; Mans and Ribeiro, 2008; Francischetti,2010
Ra-HBPs	Innate immune responses: histamine-binding Anti-inflammatory	*R. appendiculatus*	Paesen et al., 1999; Mans,2005
SHBP	Innate immune responses: histamine and serotonine-binding Anti-inflammatory	*D. reticulatus*	Sangamnatdej et al.,2002
Japanin	Immunomodulation: monocyte-derived dendritic cells, reprogrammes dendritic cell responses Immunotherapeutic (autoimmune disorders, allergies, transplant rejection, acute and chronic inflammatory diseases)	*R. appendiculatus*	Preston et al., 2013; Roversi et al.,2017
HA24	Innate immune responses: histamine-binding Anti-inflammatory	*Hyalomma asiaticum*	Wang et al.,2016
**Ixodegrin superfamily**			
Variabilin	Antiplatelet agent: inhibitor of glycoprotein IIb/IIIa (integrin αIIbβ3) Antithrombotic	*Dermacentor variabilis*	Wang et al.,1996
YY-39	Antiplatelet agent: blocks platelet adhesion to soluble collagen and bind to glycoprotein IIb/IIIa Antithrombotic	*Ixodes pacificus*	Tang et al.,2015
**Metastriate-specific families**			
Evasins	Innate immune responses: chemokine binding Anti-inflammatory (myocarditis, arthritis), anti-fibrotic	*Rhipicephalus sanguineus* and other hard ticks	Hajnicka et al., 2005; Frauenschuh et al., 2007; Deruaz et al., 2008; Hayward et al., 2017; Singh et al., 2017; Eaton et al.,2018
**Small immunoregulatory peptides**			
Hyalomin-A and -B	Innate immune responses: pro-inflammatory cytokine inhibitor Anti-inflammatory	*H. asiaticum asiaticum*	Wu et al.,2010
Amregulin	Innate immune responses: pro-inflammatory cytokine inhibitor Anti-inflammatory	*A. variegatum*	Tian et al.,2016
**∼8 kDa tick-derived C5 inhibitors**			
RaCI	Complement: inhibitor of C5 activation Anti-inflammatory	*R. appendiculatus*	Matthijs et al.,2016
**Prostriate-specific families**			
**Isac protein family**			
Isac	Complement: inhibition of the alternative complement pathway (AP) by destabilizing the C3 convertase Anti-inflammatory	*I. scapularis*	Valenzuela et al.,2000
Salp 20	Complement: inhibits the AP by binding properdin and dissociating active C3 convertase Anti-inflammatory	*I. scapularis*	Tyson et al., 2007; Hourcade et al.,2016
Irac I, II	Complement: inhibition of AP by destabilizing the C3 convertase Anti-inflammatory	*I. ricinus*	Daix et al.,2007
IxACs	Complement: inhibits the AP by binding properdin Anti-inflammatory	*I. ricinus*	Couvreur et al.,2008
**Family of 15 kDa salivary proteins**			
Salp15	Immunosuppression: multifunctional, CD4^+^ T cells, dendritic cells Immunosuppressive therapy in transplantation	*I. scapularis*	Anguita et al., 2002; Hovius et al., 2008; Tomás-Cortázar et al.,2017
**Putative secreted salivary gland proteins**			
TIX-5 (formerly P23)	Anticoagulatio: inhibitor of FXa-mediated FV activation	*I. scapularis*	Schuijt et al., 2011, 2013
**EF-hand calcium-binding proteins**			
Longistatin	Anticoagulant: plasminogen activator, degrades fibrin clots, antagonist to RAGE and suppresses inflammation during severe tissue injury Antithrombotic RAGE-regulated diseases, e.g.; Alzheimer’s disease, psoriasis, diabetic complications and tumorigenesis	*H. longicornis*	Anisuzzaman et al. (2010, 2011, 2014, 2015)
Glycine-rich, or proline-rich, collagen-like superfamily	Glycine-rich cement proteins – cement cone, prevent loss of fluids and secures tick mouthparts in the skin New medical adhesives	Various species of hard ticks	Suppan et al.,2018

*TAP, tick anticoagulant peptide; BmTI-A, *Boophilus microplus* trypsin inhibitor A; Ir-CPI, coagulation contact phase inhibitor from *Ixodes ricinus;* TdPI, tick-derived peptidase inhibitor; Iris, *I. ricinus* immunosuppressor; IRS-2, *I. ricinus* serpin 2; DsCystatin, *Dermacentor silvarum* cystatin; Ra-HBPs, *Rhipicephalus appendiculatus* histamine-binding proteins; SHBP, *Dermacentor reticulatus* serotonin-histamine binding protein; OmCI, *Ornithodoros moubata* complement inhibitor; HA, *Hyalomma asiaticum*; RaCI, *R. appendiculatus* complement inhibitor; Isac, *Ixodes scapularis* anticomplement; Irac, *I. ricinus* anticomplement; Salp, salivary protein; IxACs, *Ixodes* anticomplement proteins; YY-39, ixodegrin from *I. pacificus;* TIX-5, tick inhibitor of factor Xa toward factor V.*

The **tick anticoagulant peptide** **TAP** is the first Kunitz-domain protease inhibitor identified in tick saliva that was functionally characterized and prepared in recombinant form. It was originally isolated from the soft tick *O. moubata* (Waxman et al., 1990). TAP is a single Kunitz domain direct slow, tight-binding competitive inhibitor of FXa, with a unique binding mode and high affinity to FXa (Wei et al., 1998). Recombinant forms of TAP (rTAP) have been tested in a variety of animal models of venous and arterial thrombosis showing that the molecule was more effective than heparin and was at least as effective as hirudin, but produced less bleeding (Yeh et al., 2012). For example, in an *in vivo* study, following an infusion into rhesus monkeys rTAP inhibited generation of fibrinopeptide A induced by thromboplastin (Neeper et al., 1990). In another study, the antithrombotic effect of rTAP was tested and compared with heparin in a baboon model of arterial thrombosis. The results also demonstrated the antithrombotic effect of rTAP without alterations of primary hemostasis (Schaffer et al., 1991). In a mouse carotid artery thrombosis model, TAP-antibody targeting activated platelets fusion protein was more effective than enoxaparin without prolonged bleeding time in comparison to conventional anticoagulants (Stoll et al., 2007). These results initiated speculations that drugs targeting FXa could be safer than thrombin inhibitors, although TAP has not been tested in humans (Yeh et al., 2012). In addition, direct FXa inhibitors, including TAP, could potentially be used in prevention of other diseases, such as atherosclerosis or atrial fibrillation, because FXa as well as thrombin are involved in mediation of protease-activated receptor signaling and modulation of cellular mechanisms in the abovementioned pathophysiological processes (Spronk et al., 2014).

**Ornithodorin** from *O. moubata* was the first thrombin inhibitor identified in a soft tick. It has two domains of the Kunitz basic pancreatic trypsin inhibitor (BPTI) family. The N-terminal domain binds to the active site of thrombin, the C-terminal domain binds at the fibrinogen recognition exosite. Ornithodorin is a slow, tight-binding, competitive inhibitor of thrombin (van der Locht et al., 1996).

Another tick Kunitz domain protease inhibitor with promising antithrombotic and anti-tumor therapeutic usage, **Ixolaris,** a two-Kunitz domain inhibitor that displays homology to the tissue factor (TF) pathway inhibitor (TFPI), was obtained by screening the cDNA library derived from salivary glands of *Ixodes scapularis* (Francischetti et al., 2002, 2004). Ixolaris inhibits factor VIIa (FVIIa)/TF–induced factor (FX) activation by binding to the FXa exosite (Francischetti et al., 2002) and also binds plasmatic FX, decreases heparin-catalyzed inhibition by antithrombin III, and impairs binding of FXa to plasmatic or immobilized heparin (Monteiro et al., 2005). By using a rat model, application of ixolaris resulted in effective antithrombotic activity, without hemorrhage and bleeding (Nazareth et al., 2006). Several evidences showed close correlation between thrombosis and cancer (Timp et al., 2013). Ixolaris was shown to block TF-dependent procoagulant activity in human melanoma cell lines and inhibit their metastatic potential as well as tumor angiogenesis in mice, without evidence of bleeding (de Oliveira et al., 2012). Ixolaris was also associated with reduced tumor vascularization and expression of vascular endothelial growth factor (VEGF) in a human glioblastoma model (Carneiro-Lobo et al., 2009, 2012). All this makes ixolaris a promising agent for anticancer therapy. In a recent study, the therapeutic potential of Ixolaris in chronic infections with immunodeficiency virus (HIV) has been demonstrated (Schechter et al., 2017). Despite effective anti-HIV infection therapy, persistent inflammation involving activated monocytes and their product (e.g., soluble TF) can result in cardiovascular and thromboembolic diseases (Funderburg and Lederman, 2014). In a non-human primate model, pigtail macaques chronically infected with Simian immunodeficiency virus (SIV) exhibited high numbers of monocytes expressing TF and producing proinflammatory cytokines such as tumor necrosis factor (TNF)-α, interleukin (IL)-1β and IL-6, simultaneously leading to coagulopathy and inflammation. *In vitro*, low doses of Ixolaris inhibited TF activity (formation of FXa) in peripheral blood mononuclear cells (PBMCs) derived from chronically SIV-infected macaques and in unstimulated and LPS-stimulated PBMCs originating from anti-HIV therapy naïve and treated HIV^+^ suppressed humans, but without effect on TF expression or cytokine production (Schechter et al., 2017). *In vivo*, administration of Ixolaris to pigtails macaques showed reduced levels of IL-17 and decreased expression of Glut-1, CD80 and CD86 (i.e., markers associated with activation of lymphocytes), and resulted in lower concentration of CD4^+^ and CD8^+^ T cells (HLA-DR^+^, CD38^+^) and reduced expression of TF by CD14^+^ monocytes. In addition, Ixolaris-treated animals showed reduced plasma D-dimer levels, indicating cardiovascular comorbidities, lower SIV viremia and no developing disease up to 100 days after infection. This study has suggested a great potential of Ixolaris in anticoagulant therapy of HIV+ humans and in treatment of other inflammatory diseases (Schechter et al., 2017).

**Amblyomin-X** is a non-competitive inhibitor of FXa identified in salivary glands of *Amblyomma cajennense* (currently *A. sculptum*) ticks (Batista et al., 2010). It contains a single Kunitz domain and is able to inhibit FXa, prothrombinase and tenase activities (Morais et al., 2014; Branco et al., 2016). In an *in vitro* murine melanoma model, Amblyomin-X decreased tumor mass and reduced metastasis as well as induced apoptosis (Chudzinski-Tavassi et al., 2010; Ventura et al., 2013). It also displays cytotoxic activity on several human tumor cells, among them SK-Mel-28 (human melanoma) or Mia-PaCa-2 (human pancreatic adeno-carcinoma) cell lines, but not on non-tumor cells (Simons et al., 2011), and promotes apoptosis probably by targeting the ubiquitin-proteasome system (Chudzinski-Tavassi et al., 2010). In addition, Amblyomin-X induces apoptosis in murine renal adenocarcinoma (RENCA) cells, mitochondrial damage and the production of reactive oxygen species (ROS) (Akagi et al., 2012; Maria et al., 2013). Amblyomin-X impairs cell migration and causes actin cytoskeleton disruption in human tumor cells (Schmidt et al., 2018). In addition, tumor regression and the reduction of lung metastasis after administration of recombinant Amblyomin-X have been observed in animal models (de Souza et al., 2016). Apart its effects on tumor growth, Amblyomin-X displays antiangiogenic properties and inhibits vascular endothelial growth factor A (VEGF-A)-induced angiogenesis in both the dorsal subcutaneous tissue of mice and the chicken chorioallantoic membrane by modulation of endothelial cell proliferation and adhesion, especially of membrane expression of platelet-endothelial cell adhesion molecule-1 (PECAM-1) (Drewes et al., 2012, 2015), suggesting the possible application of Amblyomin-X as a local inhibitor to undesired neovascularization. Recently, the protein is being developed as anti-tumor drug and is under preclinical evaluations, undergoing pharmacokinetic and toxicity investigations in animals (Boufleur et al., 2019; Maria et al., 2019). These studies revealed that Amblyomin-X did not cause any mortality in mice, toxicity signs were observed only at higher doses, and there was no accumulation of Amblyomin-X in any organ.

*Rhipicephalus (Boophilus) microplus* possess a trypsin inhibitor A, **BmTI-A**, a two Kunitz domain inhibitor involved in counteracting host hemostasis. BmTI-A blocks neutrophil elastase, plasma kallikrein (Tanaka et al., 1999), trypsin and plasmin and, according to the latest information, it inhibits angiogenesis in a vessel formation assay *in vitro* (Soares et al., 2016). Neutrophil elastase is a serine proteinase secreted by neutrophils and macrophages during inflammation and destroys bacteria. It belongs to the same family as chymotrypsin and is closely related to other cytotoxic immune serine proteases, such as granzymes and cathepsin G. Abnormal expression of neutrophil elastase can cause emphysema, a chronic obstructive pulmonary disease. In elastase-induced experimental emphysema, BmTI-A minimizes parenchymal lesions in mice, suggesting the potential application of this inhibitor in emphysema treatment (Lourenço et al., 2014).

*Ixodes ricinus*- derived inhibitor of contact phase, **Ir-CPI,** with one Kunitz domain inhibits the intrinsic coagulation pathway by interference with FXIIa, FXIa and kallikrein. In addition, it protects Ir-CPI treated mice against collagen- and epinephrine-induced thromboembolism without increasing bleeding (Decrem et al., 2009).

Tick-derived protease inhibitor, **TdPI,** was identified in salivary glands of *Rhipicephalus appendiculatus* females (Paesen et al., 2007). TdPI is only expressed during the first 4 h after tick attachment and manipulates host immune defenses during the tick feeding process. It is a glycosylated Kunitz-related potent inhibitor of human β-tryptase and trypsin and moderately affects human plasmin (Paesen et al., 2007; Bronsoms et al., 2011). Human β-tryptases, specific serine proteases of mast cells, together with leukocyte elastase and chymase, are involved in inflammation and different aspects of tissue remodeling (Caughey, 2007). Beta-tryptase is a clinically useful marker of mast cells and their activation and in addition, it contributes to the pathogenesis of allergic inflammatory disorders, e.g., asthma. Thus, β-tryptase is a potential therapeutic target of tryptase inhibitors which have therapeutic potential in asthma (Sommerhoff and Schaschke, 2007). TdPI is able to penetrate mouse mast cells and may block the autocatalytic activation of tryptase required for its biological action (Paesen et al., 2007). Thus, TdPI-derived drugs could be used as inhibitors of mast cell tryptase in the control of injury caused by parasites, and in the treatment of allergies.

**Tryptogalinin**, an *I. scapularis* salivary Kunitz-type protein, was found to inhibit a number of serine proteases involved in inflammation and vertebrate immunity: β-tryptase, β-trypsin, α-chymotrypsin, plasmin, matriptase and elastase, showing a potential broad effect against mast cell proteins and other host enzymes (Valdés et al., 2013). Tryptogalinin is phylogenetically related to TdPI and provides another example when understanding of the structure and function of a tick protein could help in engineering highly specific pharmacological agents.

**Haemangin** was identified as a salivary Kunitz inhibitor in *Haemaphysalis lonigicornis* which is up-regulated during blood feeding (Islam et al., 2009). It strongly inhibits trypsin, chymotrypsin and plasmin and thereby supporting plasmin-dependent fibrinolysis inhibition and indicating its antiproteolytic potential on angiogenic cascades (Islam et al., 2009). Haemangin also blocks chick aortic explant angiogenesis and neovascularization of chick chorioalantoic membrane, demonstrating that it can inhibit both pre-existing vessel angiogenesis and neovascularization. Haemangin also impedes differentiation, proliferation, and tube formation and significantly induces expression of a variety of genes involved in apoptosis, angiogenesis and wound healing in human umbilical vein endothelial (HUVEC) cells (Islam et al., 2009).

**Disagregin** is a Kunitz-type protein derived from *O. moubata* that inhibits activation of platelet aggregation through integrin aIIbb3 (Karczewski et al., 1994). It does not contain the RGD motif that binds to the fibrinogen-binding site and thus, it is unique in its sequence as well as function and could serve to design therapeutically useful antithrombotics.

### Serine Protease Inhibitors – Serpin Domain Family

Serpins form one of the largest families of serine protease inhibitors ubiquitously distributed in nature, yet abundant in ticks (Francischetti et al., 2009). Several next-generation sequencing transcriptome studies revealed a high number of transcripts, e.g., over 150 in genus *Amblyomma*, around 20 in *Rhipicephalus* and at least 36 in *I. ricinus*, but only 20 tick serpins from different tick species have been functionally characterized. One of their functions is manipulation of host innate immune responses by impacting enzymes released from neutrophils, mast and dendritic cells (DCs).

The first tick serpin affecting host immune defenses was identified in salivary glands of *I. ricinus* and was named **Iris** (Leboulle et al., 2002). It inhibits T cells and splenocytes proliferation and alters cytokines levels of PBMC (Leboulle et al., 2002). It also suppresses coagulation and fibrinolysis by inhibition of thrombin, FXa and tissue plasminogen activator (Prevot et al., 2006). In addition, Iris inhibits secretion of TNF-α after binding on monocyte/macrophage cells (Prevot et al., 2009). Thus, Iris modulates multiple host processes simultaneously via independent mechanisms and potentially can serve for design of therapeutic for TNF-α overexpression induced pathological situations.

**FIGURE 1:**
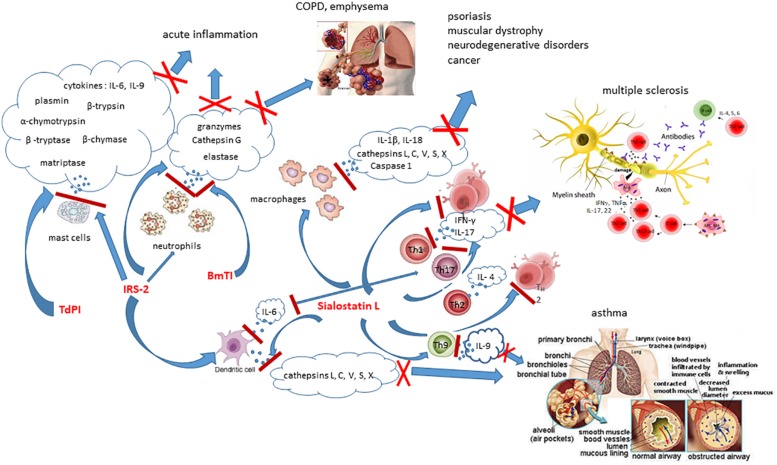
Effects of selected tick salivary proteins on host immune cells and their potential therapeutic usage to treat immunological disorders. IRS-2, BmTI, and Sialostatin L modulate functions of enzymes released by immune cells, resulting in inhibition of their biological and pathological functions.

*Ixodes ricinus* serpin 2 **(IRS-2)** is a serine protease inhibitor that specifically inhibits two proteases, cathepsin G and chymase (Figure 1) that are secreted in mammals by stimulated neutrophils and mast cells, respectively. Cathepsin G is involved in tissue remodeling during inflammation, thus IRS-2 postpones this process, as well as platelet aggregation resulting in facilitating feeding (Chmelar et al., 2011). IRS-2 also modulates production of IL-6 by DCs, and subsequently differentiation and maturation of T helper 17 cells (Th17) via the IL-6/STAT-3 (Signal Transducer and Activator of Transcription 3) signaling pathway (Palenikova et al., 2015). In addition, IRS-2 impedes the paw edema development and the influx of neutrophils in an animal model of acute inflammation (Chmelar et al., 2011).

### Cystatins (Cysteine Protease Inhibitors)

Cystatins represent a large superfamily of reversible and tight-binding inhibitors of papain-like cysteine proteases and legumains (Abrahamson et al., 2003). They regulate diverse vertebrate biological processes such as development of immune system, epidermal homeostasis, antigen presentation, neutrophil chemotaxis during inflammation, or apoptosis. Tick cystatins can interfere with both, innate and adaptive immune responses of the vertebrate host (Lieskovská et al., 2015a,b). Over the last decade, around 20 cystatins from various hard and soft ticks have been identified and biochemically analyzed for their role in the tick’s physiology and blood feeding (Schwarz et al., 2012). All these cystatins are effective inhibitors of papain-like cysteine proteases, but not of legumain and belong to two of four existing cystatin subgroups, namely type 1 cystatins/stefins) and type 2 cystatins. While stefins are known to be primarily intracellular cytoplasmic proteins, type 2 are expressed and secreted in salivary glands and midgut and therefore have been more intensively studied in ticks. **Sialostatin L** was found first in the salivary glands of *I. scapularis* ticks by a sialotranscriptome study (Valenzuela, 2002) and later **Sialostatin L2** was described (Kotsyfakis et al., 2007).

**Sialostatin L** exerts miscellaneous immunosuppressive effects on mammalians. It is a potent inhibitor of lysosomal cysteine cathepsins L, C, V, S, X (Figure 1) and papain, proliferation of CD4+ cells and cytotoxic T cells (Kotsyfakis et al., 2006, 2007, 2010; Sa-Nunes et al., 2009), migration of neutrophils during acute inflammation, secretion of cytokines by mast cells, DCs and lymphocytes, and shows preventive potential against autoimmune diseases (Kotsyfakis et al., 2006; Sa-Nunes et al., 2009). On one side, mammalian cathepsins S, L, and V represent key players in the vertebrate immunity through their participation in the antigen presentation processes of DCs and macrophages, on the other hand, they are involved in many pathological processes, such as psoriasis, muscular dystrophy, neurodegenerative disorders, cancer, etc. Sialostatin L strongly decreases the production of IL-9 by Th9 cells, essentially involved in the induction of asthma symptoms. Application of sialostatin L almost completely abrogates airway hyperresponsiveness and eosinophilia in a model of experimental asthma and experimental data suggest its preventing effect in this disease model (Horka et al., 2012), probably by suppressing mast cell-derived IL-9 production by inhibiting IRF4 (Klein et al., 2015). In a mouse model of multiple sclerosis, *in vivo* administration of Sialostatin L during the immunization phase of experimental autoimmune encephalomyelitis pointedly prevents disease symptoms associated with decreased production of IFN-γ and IL-17 and proliferation of specific T cell (Sa-Nunes et al., 2009).

**Sialostatin L2** impairs cathepsins C, L, S and V, diminishes secretion of IL-1b and IL-18 by macrophages and inhibits maturation of caspase-1. Upon infection with bacterial agents (e.g., *Anaplasma phagocytophilum*), Sialostatin L2 can significantly attenuate the severity of the disease due to its anti-inflammatory effects (Chen et al., 2014). Sialostatin L2 was found to suppress the interferon mediated immune response and enhance TBEV replication in murine DCs (Lieskovská et al., 2015a) and impair the production of proinflammatory chemokines in murine DCs upon infection with *Borrelia burgdorferi* (Lieskovská et al., 2015a).

**Iristatin,** a novel type 2 cystatin was identified in *I. ricinus* (Kotál et al., 2019). It suppresses vertebrate cathepsins C and L with similar affinity as sialostatins, production of T-cell derived cytokines, such as pro-inflammatory IFN-γ and IL-2 by stimulated Th1 cells, anti-inflammatory cytokine IL-4 by Th2 cells and IL-2 and IL-9 by Th9 cells, without effect on IL-17 production by Th17 cells. In mast cells, it blocks IL-9 and IL-6 production, but not IL-4. Furthermore, Iristatin inhibits OVA antigen-induced murine CD4+ T-cell proliferation *in vitro* and *in vivo* leukocyte (neutrophils and myeloid cells) recruitment in a mouse model of thioglycollate induced peritonitis. Iristatin with such a broad-spectrum of immunosuppressive activities may be useful in the therapy of immune-mediated diseases.

**DsCystatin** was identified by screening of the cDNA of *Dermacentor silvarum* salivary glands (Sun et al., 2018). DsCystatin impairs the activities of human cathepsins L and B and the expression of pro-inflammatory cytokines IFNγ, TNFα, and IL6 in mouse bone marrow-derived macrophages. It inhibits activation of mouse bone marrow-derived DCs and activation of NFκB in TLR2 and TLR4 signaling pathways. In a mouse arthritis model, DsCystatin suppresses joint inflammation induced by CFA and *B. burgdorferi*. DsCystatin could potentially be used in the cure of inflammatory diseases.

### Small Peptide Inhibitors of the Hirudin-Like/Madanin/Variegin Superfamily

This superfamily comprises mainly small antithrombins and is probably specific to metastriate ticks (Francischetti et al., 2009).

**Variegin** is a 32-residue peptide derived from the salivary glands of partially fed female *Amblyomma variegatum* ticks. Its C-terminal tail displays homology to hirudin and is one of the smallest thrombin inhibitors in nature (Koh et al., 2007). It is a direct thrombin inhibitor and binds to thrombin from the active site to exosite-I (Koh et al., 2011). Upon binding, variegin is cleaved by thrombin, and its cleavage product continues to inhibit the thrombin active site in a non-competitive manner (Koh et al., 2009). Variegin is structurally and functionally similar to, but is more potent than bivalirudin (hirulog), a drug used for the treatment of patients with acute coronary syndromes (Lincoff et al., 2004; Stone et al., 2006). Pharmacokinetic studies involving rats confirmed that variegin, similar to other small peptide antithrombins, e.g., bivalirudin, was rapidly excreted by the renal route, which makes it suitable for short-lasting intravenous anticoagulation during surgical procedures^1^.

Variegin-like thrombin inhibitors are probably synthesized in ticks as larger precursors that can be processed into multiple variants. Iyer et al. (2017) described **avathrin,** a new variegin-like thrombin inhibitor in *A. variegatum*. Similar to variegin, avathrin is a fast, tight-binding competitive inhibitor interacting with the thrombin active site and exosite-I after binding to thrombin. Avathrin is cleaved by thrombin, but the C-terminal cleavage product continues to exert prolonged inhibition. Avathrin prevented thrombosis better than hirulog-1 in a FeCl_3_-induced murine carotid artery thrombosis model (Iyer et al., 2017, 2019). Variegin-like peptides represent perspective candidates for designing anticoagulants to prevent arterial and venous thrombosis during invasive procedures and for coating of medical devices.

**Sculptin**, a new thrombin inhibitor was identified in the transcriptome of salivary glands of *Amblyomma sculptum* (formerly *A. cajennense*) (Iqbal et al., 2017). Scupltin consists of 168 amino acid residues, has four similar repeats, shares few similarities with hirudin, but is more similar with serine protease inhibitors of the antistasin family. Sculptin is a novel class of competitive, specific and reversible thrombin inhibitors and its mechanism of inhibition slightly differs from hirudin. Sculptin is cleaved by thrombin, but its fragments have no thrombin inhibitory activity. In contrast, fragments produced after hydrolysis by FXa are able to inhibit thrombin independently. Sculptin and its independent domain(s) have a potential to become novel antithrombotic drugs.

**Madanin-1, 2** and **chimadanin** are small, cystein-free (∼6 kDa) specific antithrombins isolated from the salivary glands of *H. longicornis* (Iwanaga et al., 2003). These peptides behave as cleavable competitive inhibitors of thrombin, bind to the active site and exosite II of the enzyme, and lose their affinity to thrombin upon proteolysis (Figueiredo et al., 2013; Thompson et al., 2017). Tyrosine sulfation of madanin-1 and chimadanin are crucial for their thrombin inhibitory activity. A dramatic increase in their potency was observed following tyrosine sulfation, with the sulfated tyrosine residues binding to exosite II of thrombin. The importance of tyrosine sulfation and the unique binding mode of these peptides make them promising candidates for the development of next generation thrombin inhibitors (Thompson et al., 2017).

**Hyalomin 1,** a 59-residue cystein-free peptide was identified in the salivary gland transcriptome of *Hyalomma marginatum rufipes*. The peptide is a selective and competitive inhibitor of thrombin, interacting with both the active site and exosite I of the enzyme (Jablonka et al., 2015). Hyalomin-1 also inhibits the thrombin-mediated activation of FXI, thrombin-mediated platelet aggregation, and the activation of FV by thrombin. It is cleaved by thrombin and cleavage region and the C-terminal fragment inhibited the enzyme similar to the full-length peptide. Testing of hyalomin-1 in a mouse model of thrombosis increased arterial occlusion time, making the peptide as another candidate for development of antithrombotic drugs.

### Basic Tail-Secreted Proteins (BTSP)

This family comprises over hundred proteins that were found primarily in *Ixodes* species (Francischetti et al., 2009; Chmelar et al., 2012). In spite some homologs from metastriate ticks were identified, BTSP seem to be typical for prostriate ticks. Most members of this protein family contain a basic carboxy terminus (tail) (Francischetti et al., 2009). Only two BTSP have been functionally characterized. They include anticoagulants with novel mode of action, derived from salivary glands of *I. scapularis*: salivary protein 14 (Salp14), and Ixonnexin.

**Salp14,** a 9.8 kDa protein, was found to impair the intrinsic pathway of coagulation and specifically inhibit FXa, but it does not inhibit other proteases (Narasimhan et al., 2002).

**Ixonnexin** is a 11.8 kDa salivary protein, displaying homology to Salp14 (Assumpção et al., 2018). It also interacts with FXa, but in addition, promotes fibrinolysis *in vitro* by enzymatically productive ternary complex through binding to tissue type plasminogen activator (t-PA) and plasminogen. In *in vivo* experiments, ixonnexin was found to inhibit FeCl_3_-induced thrombosis in mice and appears as a novel modulator of fibrinolysis that can be involved in studies on participation of plasmin in ischemic events, tumor growth and metastasis.

## Lipocalins

Lipocalins, a family of ubiquitous barrel-shaped proteins with low molecular weight, perform multiple biological functions, including the regulation of cell homeostasis and immune responses via sequestering small hydrophobic molecules such as vitamins, steroids, histamine, serotonin, prostaglandin, involved in modulation of platelet aggregation, vasoconstriction and inflammation (Schlehuber and Skerra, 2005). Lipocalins are a protein family with a large expansion in ticks; in soft ticks they act as anti-complement factors (Nunn et al., 2005; Tambourgi and van den Berg, 2014), inhibitors of platelet aggregation (Keller et al., 1993; Waxman and Connolly, 1993) and toxins (Mans et al., 2003), while in hard ticks they scavenge biogenic amines such as histamine and serotonin (Paesen et al., 1999; Sangamnatdej et al., 2002) or leukotriene B4 (LTB4) (Beaufays et al., 2008). Tick lipocalins are considered outliers since they lack the three structural conserved motifs typical of the general lipocalin family, which are apparently designed to accommodate charged, hydrophilic ligands. Unlike other lipocalins, tick lipocalins harbor two internal binding sites, the H for histamine with high affinity and the L also for histamine but weakly (Paesen et al., 2000).

**OmCI (***O. moubata* Complement Inhibitor), a 16.8-kDa protein from *O. moubata*, is the first described natural complement inhibitor from ticks, targeting specifically the C5 activation step in the complement cascade (Nunn et al., 2005; Roversi et al., 2007; Mans and Ribeiro, 2008; Barratt-Due et al., 2011).

The vertebrate complement system is a key member of innate defenses against infection, maintaining tissue homeostasis and orchestrating the crosstalk between adaptive and innate immunity (Harris, 2018). However, it is also involved in common and serious diseases, among them in many autoimmune diseases such as rheumatoid arthritis, diabetes mellitus type 1, systemic lupus erythematosus, multiple sclerosis, and myasthenia gravis (Mollnes et al., 2002). Complement can be activated by four pathways (classical, alternative, lectin, and thrombin) (Duncan et al., 2007; Thiel, 2007; Zipfel et al., 2007a,b). Although, complement association with disease has driven a boom in complement drug discovery based on natural sources, very few drugs have progressed to late-stage clinical studies due to high target concentration and turnover, unwanted side effects and lack of clarity around disease mechanism (Tambourgi and van den Berg, 2014).

**OmCI** binds directly to C5 and thus inhibits cleavage into anaphylatoxin C5a and C5b, a subunit of the membrane attack complex (MAC; C5b-9) and prevents MAC-mediated lysis and destruction of red blood cells in paroxysmal nocturnal hemoglobinuria (PNH) and tissue destruction in various other complement-mediated inflammatory and autoimmune diseases (Hepburn et al., 2007; Fredslund et al., 2008). In addition, OmCI captures the inflammatory mediator leukotriene B4, a potent chemotactic agent and activator of neutrophils (Yoshikai, 2001). A recombinant form of OmCI (known as Coversin and rEV576) has shown efficacy in numerous animal models of complement-mediated diseases and successfully accomplished a phase Ia clinical trial. The protein is protective in rat experimental models of passive and active autoimmune myasthenia gravis. OmCI-treated animals exhibited fewer symptoms and a markedly attenuated inflammatory response (Hepburn et al., 2007; Soltys et al., 2009). Coversin, if not bound to C5, has a very short plasma half-life and requires frequent dosing. N-terminal conjugation with a 600 amino acid polypeptide composed of Pro, Ala, and Ser has improved the pharmacokinetics of Coversin by extending the half-time, slowing kidney clearance, and considerably reducing background hemolysis of erythrocytes. Moreover, Coversin reduced lysis of erythrocyte as effectively as its non-conjugated form in a clinically relevant *in vitro* model of the complement-mediated disease, PNH (Kuhn et al., 2016).

**Moubatin** is a lipocalin derived from salivary glands of *O. moubata*. It inhibits platelet aggregation induced by collagen (Keller et al., 1993; Waxman and Connolly, 1993) and binds to thromboxane A_2,_ a potent platelet aggregation agonist and vasoconstrictor (Mans and Ribeiro, 2008). Moubatin was found to relax rat aorta pre-constricted by U46619 (a thromboxane A_2_ mimetic) and inhibit its contraction induced by U46619 (Mans and Ribeiro, 2008).

The hard tick *R. appendiculatus* has two types of salivary lipocalins that are structurally resolved, bind different ligands and have separate functions, namely, *R. appendiculatus*-histamine binding proteins, **Ra-HBPs** (Paesen et al., 1999) and **Japanin** (Preston et al., 2013). The high-affinity Ra-HBPs were identified in both genders of the tick: RaHBP1 and 2 are specific for females, Ra-HBP3 is associated with males. Ra-HBP1 and 2 are around 20 kDa unglycosylated monomeric proteins secreted by the tick females during the early feeding stage, while Ra-HBP3 is a glycosylated dimeric protein produced throughout feeding. Ra-HBP2 and 3 show strong affinity to histamine whereas affinity of Ra-HBP1 is weak. Ra-HBP2 has been proposed for therapeutic use since it sequesters two histamine molecules, with different affinities, thereby reducing inflammatory responses (Paesen et al., 1999, 2000).

Serotonin-histamine binding protein, **SHBP,** identified in *Dermacentor reticulatus* ticks (Sangamnatdej et al., 2002) is a lipocalin simultaneously binding serotonin and histamine. Serotonin represents a key neurotransmitter of the central nervous system and is also involved in a number of neurological disorders (Dinan, 1996). Similarly to histamine, serotonin is a key mediator of inflammation (Askenase et al., 1980) produced by mast cells and platelets.

The described histamine binding proteins and related protein family members were under investigation as potential therapeutic agents for the treatment of various diseases: Ra-HBP2 (as rEV-131) for conjunctivitis (Nuttall and Paesen, 2001b), allergic rhinitis and asthma (Nuttall and Paesen, 2001a), carcinoid syndrome and rheumatoid arthritis (Weston-Davies, 2004), Dr-SHBP for treatment of carcinoid syndrome with high production of serotonin (Schnirer et al., 2003) and post-chemotherapy emesis with nausea and vomiting due to various neurotransmitters involving serotonin. The latter treatment focused on serotonin receptor antagonists (Hesketh, 2004). In both cases, SHBP primarily targets serotonin, with secondary anti-inflammatory effects due to its histamine-binding capabilities.

In a murine allergic asthma model, the intranasal administration of Ra-HBP2 to immunized mice before antigen challenge prevented airway hyper-reactivity by 70%, and also abrogated peribronchial inflammation, pulmonary eosinophilia, mucus hypersecretion, and IL-4 and IL-5 secretion and effectively reduced airway resistance, comparable with budesonide, the conventionally prescribed corticosteroid (Couillin et al., 2004). In a corticosteroid-resistant LPS-induced murine model of acute respiratory distress syndrome, rEV-131 decreased bronchoconstriction, activation and influx of neutrophils. In phase I and II clinical trial this lipocalin showed safety and pharmacological activity in treatment of allergy in humans (Schlehuber and Skerra, 2005).

**Japanin** is a 17.7 kDa *N*-glycosylated lipocalin which exists in complex with cholesterol (Preston et al., 2013; Roversi et al., 2017). It reprograms DCs, antigen-presenting cells, so they no longer respond to a wide spectrum of stimuli *in vitro* specific for immune recognition. DCs, resident cells within most peripheral tissues (including skin), represent a bridge between innate and adaptive immunity as the important initiators and modulators of T cell responses. Japanin blocks differentiation of DCs from monocytes and on one side impedes upregulation of co-stimulatory molecules and pro-inflammatory cytokines in response to stimuli and on other side it promotes upregulation of co-inhibitory molecules and the anti-inflammatory cytokine IL-10 (Preston et al., 2013). Although the exact mechanism by which Japanin modulates DCs has not been fully described, the molecule may represent a novel tool to modulate DCs with possible therapeutic applications, such as prevention or treatment of transplant rejection or autoimmune diseases.

**HA24,** identified in the salivary glands of the tick *Hyalomma asiaticum*, represents a new lipocalin protein with particular histamine binding capacity (Wang et al., 2016). Its recombinant form binds specifically to histamine in a dose-dependent manner, and can provide palliation from allergic asthma in mice (Yanan et al., 2016).

## Ixodegrin Superfamily

Ixodegrins include cysteine-rich proteins containing the RGD (Arg-Gly-Asp) or KGD (Lys-Gly-Asp) motif that interfere with fibrinogen binding to platelets and exert antiplatelet activities (Francischetti et al., 2009). As GPIIb/IIIa inhibitors are widely used clinically during coronary interventions (Hashemzadeh et al., 2008), tick-derived disintegrins could also serve as candidates for drug design.

**Variabilin,** derived from salivary glands of *Dermacentor variabilis*, is 5-kDa RGD-containing disintegrin and was the first RGD-containing antagonist isolated from ticks (Wang et al., 1996). It blocks ADP-induced platelet aggregation and prevents integrin aIIbb3 binding to immobilized fibrinogen. It also an antagonist of the vitronectin receptor αvβ3 and attenuates osteosarcoma cell adhesion to vitronectin. Its sequence differs from other known naturally occurring antagonists of GPIIb-IIIa.

Ixodegrins identified in *Ixodes pacificus* and *I. scapularis* salivary gland transcriptomes display sequence homology to variabilin, including the RGD domain (Francischetti et al., 2005, 2009). Using the sequence of ixodegrin in the *I. pacificus* transcriptome (Francischetti et al., 2005), the molecule **YY-39** was synthesized and refolded for studies on its effects on platelets and thrombosis *in vivo* (Tang et al., 2015). YY-39 decreased adenosine diphosphate (ADP)-, thrombin- and thromboxane A_2_-induced platelet aggregation. In addition, it inhibited platelet adhesion to soluble collagen and bound to purified GPIIb/IIIa. YY-39 also reduced thrombus weight in an *in vivo* experimental rat arteriovenous shunt model and as well as blocked thrombosis in a carrageenan-induced mouse tail thrombosis model. However, in the tested animal models, YY-39 showed little bleeding complication, which makes the molecule a promising antithrombotic agent.

## Metastriate-Specific Families

Over thirty families of proteins have been found exclusively in metastriate ticks (Francischetti et al., 2009), but only a few have been functionally characterized. These include small immunoregulatory proteins that modulate vertebrate immune responses, mainly acting on cytokines and chemokines.

Cytokines, a diverse group of small secreted proteins (interleukins, growth factors, chemokines), represent key humoral controllers of cells in their interactions and communications and regulators of processes under normal, developmental and pathological conditions (inflammation, wound healing). Cytokine through binding to their specific receptors orchestrate immune responses, including the recruitment of immune cells into the site of inflammation. Uncontrolled expressions of cytokines are associated with inflammatory, autoimmunity even with cancer, thus members of cytokine networks are targets for development of new effective therapeutics. Ticks either suppress production of cytokines by various immune cells or directly inhibit cytokines by binding ligands. However, from all anticytokine activities described in different tick species (Hajnická et al., 2001; Hajnicka et al., 2005, 2011; Vančová et al., 2007, 2010; Peterková et al., 2008; Slovák et al., 2014), only hyalomins (Wu et al., 2010), Amregulin (Tian et al., 2016) and chemokine binding factors named Evasins derived from *Rhipicephalus sanguineus* (Frauenschuh et al., 2007; Deruaz et al., 2008) have been functionally and structurally characterized.

### Small Immunoregulatory Peptides

**Hyalomin-A and -B** were identified in salivary glands of the hard tick *Hyalomma asiaticum asiaticum* (Wu et al., 2010). Both immunosuppressant peptides exert significant anti-inflammatory activities, either directly by inhibiting LPS-induced production of inflammatory cytokines – TNF-α, monocyte chemotactic protein-1and IFN-γ in mouse splenocytes or indirectly by stimulating secretion of immunosuppressant cytokine IL-10. Moreover, both hyalomins scavenge free radical 2,2′-azinobis 3-ethylbenzothiazoline-6-sulfonic acid (ABTS^+)^ radicals *in vitro* and inhibit adjuvant-induced arthritis in mice *in vivo*.

Tian et al. (2016) structurally and functionally characterized a small immunosuppressant peptide **Amregulin** from *A. variegatum*. Amregulin inhibits *in vitro* secretion of inflammatory cytokines – TNF-α, IL-8, IL-1 and IFN-γ by LPS stimulated rat splenocytes in a dose-dependent manner. Like hyalomins, this peptide shows concentration dependent antioxidant activity by scavenging free radical ABTS^+^, but not 2,2-diphenyl-1-picrylhydrazyl (DPPH) and by reducing ferric ions (Fe^3+^) to Fe^2+^. Amregulin also significantly inhibits Freuds adjuvant-induced paw inflammation in mouse models *in vivo*.

### Evasins

Three salivary glycoproteins, found in the brown dog tick *R. sanguineus*, are secreted during a feeding and bind to host chemokines, thus inhibiting the host inflammatory response (Frauenschuh et al., 2007; Deruaz et al., 2008). **Evasin-1** and **-3** are high specific binders of only three CC chemokines (CCL3, CCL4, and CCL18) and a subset of CXC chemokines (CXCL1, -2, -3, -5, -8), respectively, whereas **Evasin-4** demonstrated more promiscuity effect by interaction with at least 18 chemokines of the CC subfamily. Evasin-1 in a dose-dependent manner reduced CCL3-induced influx of neutrophils in a peritoneal cell recruitment assay and showed high efficiency in reducing fibrosis resulted from neutrophil infiltration into the lung after bleomycin treatment, and also decreased the mortality observed in this model (Russo et al., 2011). Evasin-3 is effective in several neutrophil-dependent disease models with regards to its *in vitro* effect mentioned above. Montecucco et al. (2014) demonstrated beneficial effects of Evasin-3 and partially of Evasin-4 on inflammatory processes in pancreas and lungs in a mouse model of acute pancreatitis (AP). Evasin-3 treatment of mice with cerulein-induced AP decreased recruitment of neutrophils, production of ROS and apoptosis in the lungs and inhibited necrosis and apoptosis of neutrophils and macrophages in the pancreas. Evasin-4 only decreased the abundance of macrophages in lungs, without any effects in the pancreas. A dose-dependent Evasin-3 inhibition of CXCL8 induced leukocyte infiltration into the peritoneal cavity is determined. In an antigen-induced arthritis by BSA, intradermal injection of Evasin-3 significantly suppresses disease symptoms. In case of ischemic reperfusion injury, Evasin-3 showed higher efficiency than Evasin-1, even though only Evasin-1 effectively inhibited first DCs recruitment to the site of infection with *Leishmania major*, mediated by CCL3 released from neutrophils (Charmoy et al., 2010). Moreover, Evasin-1 is able to reduce skin inflammation observed in D6−/− mice in response to 12-*O*-tetradecanoylphorbol-13-acetate (Deruaz et al., 2008). Castor et al. (2010) studied effects of Evasin-1 on pathogenesis of acute graft-versus-host disease (GVHD) in mice, induced by transplantation of C57BL/6J murine splenocytes to B6D2F1 mice. GVHD is a complication occurring in allogeneic bone marrow transplantation with significant morbidity and mortality in humans (Ferrara and Deeg, 1991), and is characteristic by CCL3 expression and CCL3-induced recruitment and proliferation of T cells. Although application of Evasin-1 did not interfere with GVH-leukemia in mice, Evasin-1 treatment reduced levels of IFN-γ and CCL5, but not TNF-α, reduced the number of CD4^+^ and CD8^+^ T cells infiltrating the small intestine and the damage of intestine and liver. In addition, Evasin-1 also ameliorated GVHD and provided partial relief from symptoms, whereby the effect was comparable to glucocorticosteroid dexamethasone and was similar to protection observed in CCL3^–/–^ mice. Furthermore, both Evasin-3 and -4 effectively reduce post-infarction myocardial injury and remodeling (Braunersreuther et al., 2013) and Evasin-4 is effective in DSS-induced colitis (Vieira et al., 2009). Because of its broad CC chemokine-binding spectrum, Evasin-4 is considered the most suitable candidate for design of a therapeutic agent. By sequence similarity searches in transcriptomes of hard ticks, mainly of the *Rhipicephalus*, *Amblyomma*, and *Ixodes* genera, over 250 putative evasin sequences were detected (Hayward et al., 2017). Eight of them were expressed in *Escherichia coli* and exhibited high-affinity binding to human chemokines. They were classified into C8 and C6 evasins. By using the yeast surface display method, Singh et al. (2017) detected ten novel polyvalent CC-chemokine binding evasin-like molecules from eight hard tick species of the *Rhipicephalus* and *Amblyomma* genera. One of them, P672 from *Rhipicephalus pulchellus*, was found to bind to CCL8 and its properties could be altered by homology modeling, demonstrating that the function of tick evasins can be manipulated to design novel drugs (Eaton et al., 2018).

### ∼8 kDa Tick-Derived C5 Inhibitors

**RaCI** (Rhipicephalus appendiculatus C5 Inhibitor) belongs to a novel protein family of ∼8 kDa tick-derived C5 inhibitors. It was identified in the transcriptome of *R. appendiculatus* salivary glands (Matthijs et al., 2016) and its sequence shares no similarity to previously characterized tick complement inhibitors. RaCI binds human C5 and blocks the generation of C5a and membrane attack complex formation. RaCI exhibits cross-species reactivity which can be used in disease models on animal to test therapeutic efficacy of drug candidates. The small size of RaCI in comparison with the previously described OmCI and retaining its potency after its further reducing may potentially aid drug administration. As OmCI and RaCI target different sites on C5 (C5d-CUB and C5d-MG1-MG2, respectively), RaCI may be used to tune therapeutic effects of complement 5 inhibitors.

## Prostriate-Specific Families

Out of the six protein families included in this group, only members of one group, i.e., the Isac protein family, have been characterized functionally. This group comprises salivary anti-complement proteins derived from *I. scapularis* and *I. ricinus* (Francischetti et al., 2009).

### Isac Protein Family

Several tick saliva molecules with promising anti-complement activities have been identified and characterized in ticks of the *Ixodes* genus: **Isac** and **Salp20** in *I. scapularis* (Valenzuela et al., 2000; Tyson et al., 2007), **Irac I,** **Irac II** (Daix et al., 2007), and **IxACs** (Couvreur et al., 2008) in *I. ricinus.* Isac and Irac proteins specifically inhibit the alternative pathway (AP) of complement via blocking C3 convertase, whereby this inhibition can lead to immunosuppression or diminishing of opsonization. On the other hand, depletion of serum C3 detected in neuromuscular junction, an important factor determining severity of myasthenia gravis, is appreciated in some patients with this autoimmune neuromuscular transmission diseases. Salp20, displaying homology to Isac, inhibits the AP by binding properdin, which is a positive regulator of this pathway (Tyson et al., 2008; Hourcade et al., 2016). Couvreur et al. (2008) cloned and expressed proteins from *I. ricinus*, named IxACs, showing 40% identity to Isac and Irac. IxACs also inhibit the AP by binding to properdin.

### Family of 15 kDa Salivary Proteins

**Salp15,** a 15 kDa immunosuppressive cystein-rich glycoprotein, was identified in salivary glands of *I. scapularis* (Anguita et al., 2002). It inhibits directly the activation of CD4+ T cells through binding on the coreceptor CD4, which results in signaling pathway inhibition, reduced IL-2 production and CD25 (IL-2Rα) expression (Juncadella et al., 2007; Garg et al., 2014), and also indirectly via inhibition of DCs functions by binding on lectin type. Salp15 binding to CD4 is persistent and induces a long-lasting immunomodulatory effect, probably due to induction of an increased expression of the ectoenzyme, CD73, in regulatory T cells and increased production of adenosine (Tomás-Cortázar et al., 2017). This immunomodulatory protein is a promising candidate for treatment of T-cell-mediated autoimmune diseases involving asthma or allogeneic transplant tolerance. Paveglio et al. (2007) tested Salp15 in a mouse model of human allergic asthma with typical imbalance of CD4+ T cells toward Th2 cells and overproduction of cytokines IL-4, IL-5, and IL-13, leading to production of IgE, eosinophilpoiesis, production and secretion of mucus. Mice were intra-peritoneally sensitized with OVA in aluminum hydroxide in combination with or without Salp15, followed by OVA-aerosol treatment. Salp15 significantly reduced all features of allergic asthma mentioned above thus effectively prevented the development of experimental asthma. An opposite effect of Salp15 was detected in a mouse model of multiple sclerosis, experimental autoimmune encephalomyelitis (EAE), the progression of which is directly associated with Th17 and Th1 cells secreting IL-17 and IFN-γ, respectively (El-behi et al., 2010). Surprisingly, application of Salp15 led to enhanced activation of Th17 cells and consequently to increased production of IL-17 and development of severe EAE in mice *in vivo* and to induced differentiation of Th17 cells with IL-6 and without TGF-β *in vitro*. Salp15 did neither affect infiltration of T cells to the central nervous system, nor the development of antibody responses against the eliciting peptide PLP_139__–__151_ or the presence of IFN-γ in the sera. The reported effect can be associated with repression of IL-2 during T cell differentiation, which could be achieved also by TGF-β and/or other immunosuppressants (Juncadella et al., 2010).

### Putative Secreted Salivary Gland Proteins

**TIX-5** (‘Tick Inhibitor of factor Xa toward factor V,’ formerly P23) was originally identified as a salivary antigen by screening *I. scapularis* nymphal salivary gland yeast surface display library with nymph-immune rabbit sera (Schuijt et al., 2011). Recombinant P23 demonstrated anti-coagulant activity. The anticoalgulant properties of P23 renamed to TIX-5 were further characterized (Schuijt et al., 2013). The studies show that TIX-5 specifically inhibits FXa-mediated factor V (FV) activation involving the B-domain of FV and reveal that activation of FV by FXa is a crucial event in the initiation of thrombin generation. The data not only elucidate a unique molecular mechanism by which ticks inhibit host blood coagulation, but propose a revised blood coagulation scheme in which direct FXa-mediated FV activation occurs in the initiation phase of coagulation. These findings could potentially results in novel therapeutic approaches for anticoagulation (Aleman and Wolberg, 2013).

### EF-Hand Calcium-Binding Proteins

**Longistatin** was the first EF-hand calcium-binding protein identified and characterized from salivary glands of an ixodid tick, *H. longicornis* (Anisuzzaman et al., 2010). The protein was found to function as an anticoagulant and plasminogen activator, hydrolyze fibrinogen and delay fibrin clot formation (Anisuzzaman et al., 2011). Moreover, longistatin modulates host immune responses and inflammation associated with tick bites by binding to the receptor for advanced glycation end products (RAGE) that mediates immune cell activation and is highly expressed in the host skin at inflammatory sites (Anisuzzaman et al., 2014). Due to its ability to block RAGE, longistatin may be a therapeutic tool against RAGE-regulated diseases such as Alzheimer’s disease, psoriasis, diabetic complications and tumorigenesis (Anisuzzaman et al., 2015).

## Glycine-Rich, or Proline-Rich, Collagen-Like Superfamily

This superfamily of proteins produced in tick salivary glands is subdivided to a number of subgroups and includes, e.g., cuticle proteins, collagens, small and large GGY peptides, metastriate spider-like cement protein, Ixodes-specific collagen-like small peptides, metastriate and argasidae proteins distantly related to Ixodes collagen-like proteins, etc. (for details see Francischetti et al., 2009). Of interest to medical applications are proteins associated with tick cement. The main function of the cement is to anchor mouthparts of the feeding tick to the host skin and seal the feeding lesion during attachment. There are important differences in the strategy of attachment of metastriate and prostriate ticks as well as in the ultrastructure, mechanical properties and chemical composition of their cement (for review see Suppan et al., 2018). Metastriate ticks having shorter mouthparts produce an abundant cement protein cone, whereas prostriate ticks have longer mouthparts that allow mechanical attach into the host dermis, and thus produce less cement.

Considering the adhesive and sealing properties of tick cement, its potential applications as a template for biomimetic tissue adhesives is proposed (Suppan et al., 2018). Currently available tissue glues contain toxic substances, have weak bonding forces, and/or do not cover all possible fields of application in surgery and microsurgery (for review see Suppan et al., 2018). However, to be able replace currently used tissue glues by adhesives developed based on the structure and function of tick cement, the adhesive molecules produced in tick salivary glands need to be further explored.

## Conclusion

The mammalian hemostatic and immune systems represent robust complex networks, comprising diverse humoral and cellular biological structures and processes that are essential for protection against disease, injuries or any other potentially damaging disturbers. Hemostasis belongs to the first line of defense and prevents from blood loss after damage of blood vessels. Functioning properly, both the hemostatic and the immune system identify a variety of threats and the immune system distinguishes them from the own healthy tissue. Hemostatic and immune pathways are closely interconnected. Any disorders of the immune system can result in immunodeficiency, autoimmune diseases, inflammatory diseases, even cancer, in which events of innate and adaptive immune responses, but also coagulation factors participate. Current treatment options of such diseases have often limited effectiveness, are accompanied by many side effects or are insufficient. Therefore researchers are looking for alternative medicaments derived from nature, for example, from plants, animals, or microorganisms. Ticks are astounding “pharmacologists.” Their saliva contains hundreds of proteins and low molecular weight effectors with immunomodulatory, anti-inflammatory, anti-clotting and anti-platelet as well as antitumor and antiangiogenic properties with high affinity, avidity and selectivity for their targets in the host defense mechanisms. Moreover, many of the tick-derived molecules show specific, yet unknown functions and modes of action. Thus, ticks are promising sources for discovering and designing new medical treats targeting various pathways of the mammalian physiology. In addition to their high target specificity, tick-derived products have a low risk of microbial resistance, toxicity and immunogenicity (Simons et al., 2011). However, the exploration and exploitation of the pharmacological potential of ticks is still in its infancy. The candidates for designing novel drugs mentioned in this study have been tested in various animal models of human diseases and have shown promising continues. Unfortunately, only a few of them advanced to pre-clinical investigations, e.g., TAP, Ra-HBP, Dr-SBP, OmCI, or Amblyomin-X. The tick lipocalin RaHBP exhibited high ligand specificity and affinity and data from clinical trial exhibited beneficial effects in human allergy. Lipocalins are promising new therapy candidates based on their natural ligand-binding functions to store and transport small compounds, which together with their structure (small polypeptide chain) led to design of artificial binding proteins “anticalins” with novel specificities, for example as transporters for pharmaceuticals. In general, it is a very long way from basic research to translation to the clinical praxis and, according to Murfin and Fikrig (2017), with many gaps and lacks. It is known that arthropod molecules are unstable, have a short half-life (for example OmCI mentioned in this review) or are cytotoxic, thus they require improved pharmacokinetics, optimization of dosage, delivery way, etc. (Ratcliffe et al., 2014). Another complication in drug development based on tick molecules is the identification of the mode of action of individual molecules within the complex mixture of saliva, where synergistic effect of molecules is presumed in the context of tick feeding. Thus, for therapeutic benefit identification of the interacting partners would be necessary. Last but not least, the high costs of development of new drugs designed on the basis of tick molecules is very relevant, thus the approval processes are not very attractive. However, only profound basic studies of the biological functions of tick molecules can lead to discovery and approval of new therapeutic agents.

## Author Contributions

PB, IŠ, VH, and MK conducted the literature search and wrote the manuscript. MK prepared the table. IŠ prepared the figure. All authors critically read and revised the manuscript.

## Conflict of Interest Statement

The authors declare that the research was conducted in the absence of any commercial or financial relationships that could be construed as a potential conflict of interest.

## Footnotes

1 https://patents.google.com/patent/US9217027
